# Characterization data of cellulose modified by gamma irradiation to be used as template in the synthesis of a photoactive composite for environmental applications

**DOI:** 10.1016/j.dib.2022.108277

**Published:** 2022-05-15

**Authors:** Laria Rodríguez-Quesada, Aura Ledezma-Espinoza, Esteban D. Avendaño-Soto, Ricardo Starbird-Perez

**Affiliations:** a Master Program in Medical Devices Engineering, Instituto Tecnológico de Costa Rica, Cartago 159-7050, Costa Rica; b Centro de Investigación en Servicios Químicos y Microbiológicos (CEQIATEC), Escuela de Química, Instituto Tecnológico de Costa Rica, Cartago 159-7050, Costa Rica; c Centro de Investigación en Ciencia e Ingeniería de Materiales (CICIMA), Universidad de Costa Rica, 11501-2060 San José, Costa Rica; d School of Physics, Universidad de Costa Rica, 11501-2060 San José, Costa Rica; e Doctorado en Ciencias Naturales para el Desarrollo (DOCINADE), Instituto Tecnológico de Costa Rica, Universidad Nacional, Universidad Estatal a Distancia, Costa Rica

**Keywords:** Functionalized cellulose, Photocalyst, Gamma irradiation, Photoactive composite

## Abstract

The following data provide evidence of the green functionalization process of a cellulose substrate by gamma radiation to be used as template in the preparation of photocatalyst composites. Functionalized cellulose, by gamma radiation treatment, improved its stability in water and exhibited a reduced size. Our data showed an intensification of carbonyl groups signal and a decrease in the thermal stability of the cellulose as result of the gamma radiation dose. Infrared and thermal data of the treated cellulose provide evidence of bond scission and the formation of functional groups that improved it is application as template. Finally, the conductive polymer poly(3,4-ethylenedioxythiophene) was deposited on the gamma irradiated cellulose to be used as photo-catalyze in the treatment of contaminated water with pharmaceutical compounds.

**Specifications Table**

**Table:** 

Subject	Materials Science, Material Characterization
Specific subject area	The data correspond to the characterization of a modified biopolymer employed as template in the preparation of a conductive composite particle.
Type of data	Tables
How the data were acquired	Instruments: 1. Fourier transformed infrared spectroscopy Nicolet 380 with an iATR accessory (Thermo Scientific, Madison, Wisconsin, USA). 2. Thermogravimetric analyser SDT Q600 from TA Instruments (New Castle, Delaware, USA). Conditions: 1. FTIR: Dry samples were placed directly onto the diamond window (ca. 2 mg) without further preparation. Measurements were made in absorbance mode, in the 4000–600 cm−1 spectral range using 32 scans at a resolution of 4 cm–1. 2. Thermal characterization was carried out in a nitrogen atmosphere (100 mL/min) with a scan rate of 10 °C/min, from room temperature to 700 °C in alumina cups (110 µL) Software: 1. OMNIC v9.3.30 2. TRIOS 5.0
Data format	Raw
Description of data collection	Avicel® microcrystalline cellulose particles (Cell) were dispersed in water and then placed in screw-cap glass vials to be irradiated using a gamma cell irradiator with a Cobalt-60 source (Ob-Servo Ignis, IZOTOP, Budapest, Hungary). Gamma doses up to 300 kGy were applied to the dispersions. The estimated error of the absorbed dose rate is 2.4% considering a 95% confidence level.
Data source location	Costa Rica, Cartago 159–7050, Instituto Tecnológico de Costa Rica.
Data accessibility	Repository: Mendeley Data Data identification number: 10.17632/m2krns7nbf.1 Direct URL: https://data.mendeley.com/datasets/m2krns7nbf/1 Data is available under the Creative Commons BY-NC-SA 4.0 license.
Related research article	A. Ledezma-Espinoza, L. Rodríguez-Quesada, M. Araya-Leitón, E. D. Avendaño-Soto, and R. Starbird-Perez, Modified cellulose/poly(3,4-ethylenedioxythiophene) composite as photocatalyst for the removal of sulindac and carbamazepine from water, Environmental Technology & Innovation, 27 (2022) 102,483. 10.1016/j.eti.2022.102483

## Value of the Data

• Gamma radiation affects the cellulose properties, increasing the oxidation level and decreasing the particle size. These irradiation dosages may be used to tune the properties of the cellulose for specific applications.
• The degradation of polymers by gamma radiation promotes reproducibly and quantitatively changes without the introduction of chemical reagents.
• Our data are useful for researchers in the area of natural, renewable, biocompatible and biodegradable polymer due to the potential use to a wide variety of applications ranging from paper products, electronics, drug coatings, food packaging, energy storage and supercapacitors.
• The data provide a hint of how the properties of the cellulose are affected by high gamma dosages during sterilization process. The oxidation of the backbone along with its breakdown may be used to tune the cellulose properties for further applications.
• Irradiated cellulose offers an outstanding template for the preparation of a photoactive composite to be used, but not limited, in environmental applications.

## 1 Data Description

**Fig. 1:**
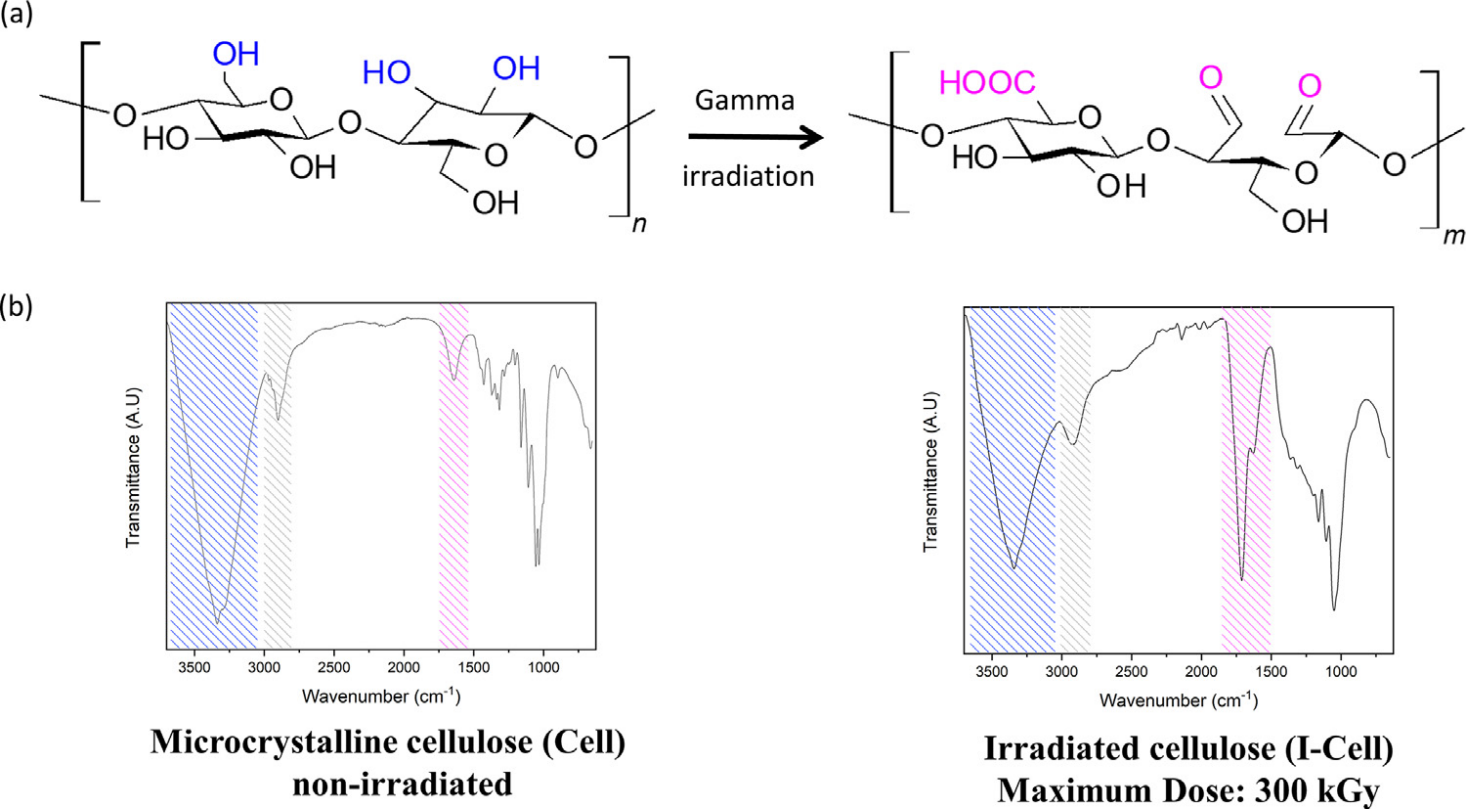
Cellulose γ-irradiation treatment: (a) schematic representation of the oxidation of the backbone process and (b) oxidation process measured by FTIR before irradiation (left) and after 300 kGy dose (right).

The collected data summarize the effect of gamma irradiation in the cellulose. The results draw attention because gamma radiation is a common tool used for sterilization [1] and it may change cellulose properties including viscosity [2], mechanical and barrier properties [3], molecular weight [4], surface area [5] and crystallinity [5]. Accordingly, the gamma effect on the physical and chemical properties may provide a feasible method to tailor the properties of the cellulose for the particular application [6]. The experimental conditions and radiation dosage induce scission or crosslinking reactions in polymers during the ionizing treatment [7]. Degradation of the cellulose, cause by the glycosidic bond splitting and oxidation (see Fig. 1a), may drive to reduce its size and generate a larger surface area [5]. Our data confirm the molecular changes on the cellulose during ionizing processes (e.g. sterilization) as seem in Fig. 1b and that those doses may be used to tune the properties of the cellulose for specific applications. The resulted irradiated cellulose was successfully used as template for the chemical deposition of poly(3,4-ethylenedioxythiophene) (PEDOT) on its surface.

### 1.1 Data of cellulose particles functionalization by gamma radiation

Infrared spectroscopy is a common and powerful technology in the chemistry field, it is especially important in the study of organic molecules. The absorption by the molecule at different frequencies, as the typical infrared, gives information about the composition, structure or stereo-regularity in the polymer chain (see Fig. 1). Some accepted signals of the cellulose are summarized in the Table 1, which shows the usefulness of the technique in the identification of the oxidation process.

**Table 1:** Infrared frequency bands assignments for cellulose (Cell) and irradiated cellulose (I-Cell).

Wavenumber (cm^−1^)	Activity^1^	Description of signals	Reference
**Experimental**			
3330	S	Hydrogen bond OH group	[4,[8], [9], [10]]
2924	W	CH stretching	[8], [9], [10]
1740	M	C=O group	[4,9]
1645	M	Polymer bound water	[9,10]
1452		CH asymmetric deformation	
1160	W	C-O-C (asymmetric stretching)	[9,11]
1049	M	C-OH bending vibration	[10,12]

1 S: strong, M: medium and W: weak.

The FTIR data showed absorption bands at 3330 and 2895 cm^−1^, related to the hydroxyl (OH) (Fig. 1b blue region) and aliphatic (C-H) stretches (Fig. 1b gray region), respectively. In addition, the peaks at 1645 and 1740 cm^−1^ were assigned to stretch carbonyl group (C=O) (Fig. 1b red region), according to previous reports [4]. The absorption signals around 1452 and 1320 cm^−1^ were assigned to the C-H flexion and the C-C stretch. The absorbance at 1740 cm^−1^ (stretching vibration C=O) caused by the oxidation of the polymer backbone [4], was normalized using the C-H band at 2924 cm^−1^ for each gamma doses (Table 2). In the irradiated samples, there is an increase in the carboxyl/aliphatic ratio as result of cellulose degradation [4]. Table 2 shows the oxidation of the cellulose due to the gamma irradiation.

**Table 2:** Cellulose oxidation ratio as a function of the applied gamma doses, measured by FTIR.

Gamma dose (kGy)	Ratio ${}^{1735\;cm^{-1}}{/}_{2990\;cm^{-1}}$
0	1.71
20	2.05
50	2.13
100	2.79
200	6.76
300	11.57

**Fig. 2:**
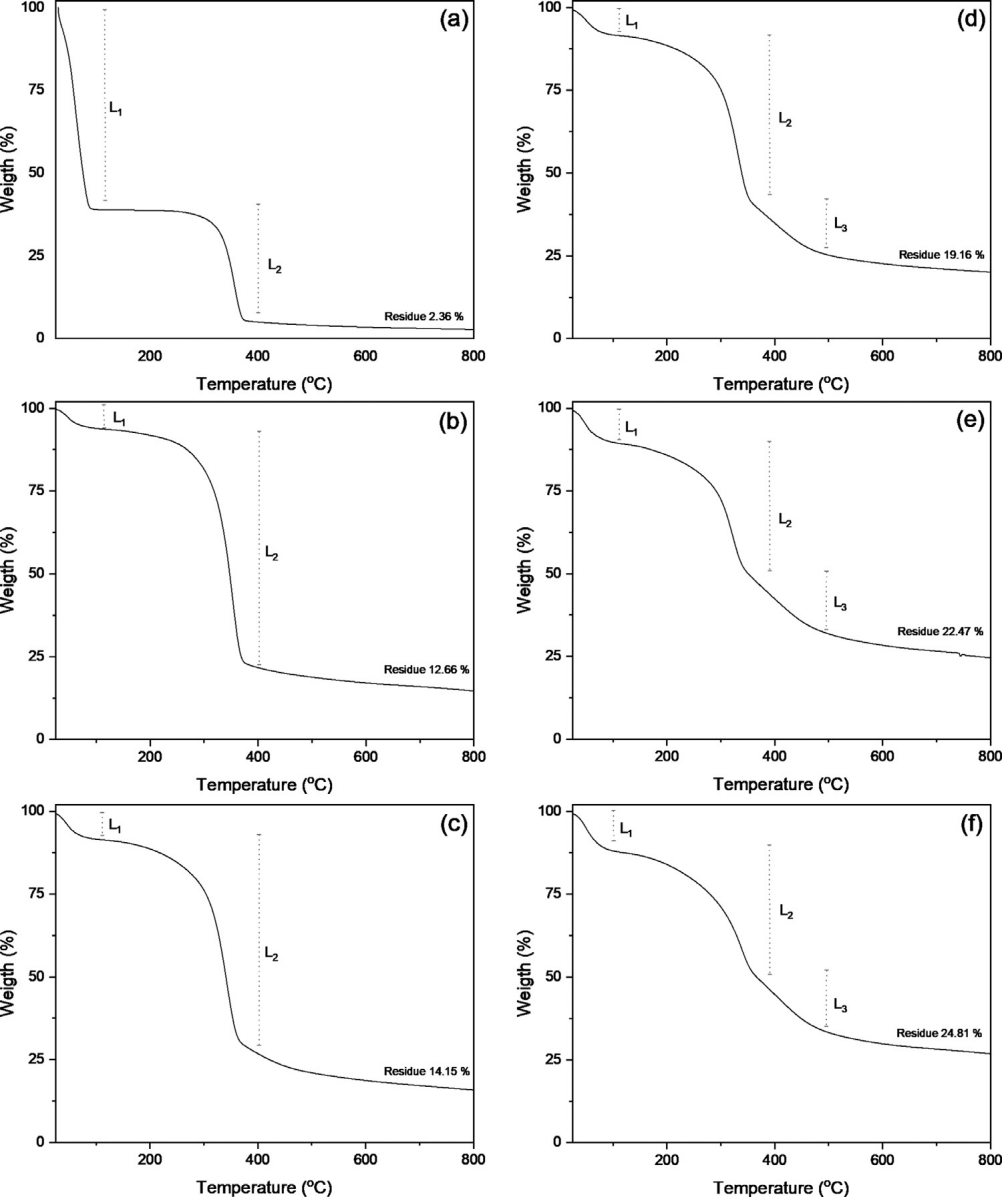
Thermal effect on cellulose due the gamma irradiation measured by means of thermogravimetric analysis: (a) blank sample (without gamma doses), (b) 20 kGy, (c) 50 kGy, (d) 100 kGy, (e) 200 kGy, (f) 300 kGy.

In order to confirm the cellulose degradation, thermogravimetric analysis (TGA) data is added. TGA is considered a robust technique for determining composition of lignocellulosic biomass and changes in its physical and chemical properties [13]. The data of the non-irradiated and irradiated sample are shown in the Fig. 2. The first loss of mass in the TGA curves is seem below 115 °C (L_1_), and it is related to the physically adsorbed water in the samples, because the biomass is stable up to 140 °C [14]. The second decomposition process (L_2_), in the range of 150 and 350 °C, is linked with cellulose degradation, specifically the alkyl ether bonds. Finally, the cellulose depolymerization occurs in the temperature range of 372 to 570 °C (L3), due to the carbon-carbon bond cleavage in the cellulose backbone [15]. Depolymerization of the cellulose occurs when it has absorbed enough energy to break of the glycosidic bond [16].

The data of the irradiated samples using gamma doses higher than 100 kGy evidenced a lower thermal stability in contrast to the non-irradiated substrate. The components of the cellulose sample gradually suffer chain breakage when the absorbed radiation dose reaches 100 kGy or higher [17]. Additionally, it is observed an increase in the residues at different irradiated doses, probably related to the backbone dehydration of the cellulose during the gamma treatment.

Our data are useful for researchers in the area of natural, renewable, biocompatible and biodegradable polymer because its potential application to a wide variety of applications ranging from paper products, electronics, drug coatings, food packaging, energy storage and supercapacitors. The data show how gamma radiation affects the cellulose properties increasing the oxidation level and decreasing the thermal stability. The degradation of polymers by gamma radiation promotes reproducibly and quantitatively changes, without the introduction of chemical reagents. In our work, irradiated cellulose provided an excellent template for the generation of a photoactive composite to be used in the environmental applications.

## 2 Experimental Design, Materials and Methods

Fourier transformed infrared spectroscopy (ATR-FTIR) was performed by means of a Nicolet 380 spectrometer with an iATR accessory (Thermo Scientific, Madison, Wisconsin, USA) and the data was treated using the OMNIC v9.3.30 software. Briefly, treated and untreated samples (ca. 2 mg) were measured without further preparation. FTIR spectra, in the 4000–600 cm−1 range, were obtained in transmittance mode. Oxidation ratio because of the gamma doses was measured using the ratio of the carboxyl/carbonyl signal (1735 cm-1) normalized by the aliphatic C-H stretching band (2990 cm-1). Data in the repository correspond to the infrared absorption data of each sample. The data is attached in CSV format for complementary analysis.

Thermal stability of the commercial and irradiated samples was measured by thermogravimetric analyses (TGA) in a SDT Q600 device (TA Instruments, New Castle, Delaware, USA). A nitrogen stream (100 mL/min) was used to maintain a reductive atmosphere during the analysis with a scan rate of 10 °C/min up to 700 °C. Data of the decomposition process (i.e. weight loss (L) and residues) were determinate for each sample. Additionally, data in the repository correspond to the Weight loss (mg), Heat Flow (mW), Temperature Difference (°C), Sample Purge Flow (mL/min) and the weight derivate (%/°C) of each sample in CSV format. The raw data may be used for further analysis regarding cellulose thermal stability.

## CRediT authorship contribution statement

**Laria Rodríguez-Quesada:** Conceptualization, Methodology, Software, Investigation, Data curation, Formal analysis, Validation, Visualization, Writing – original draft, Writing – review & editing. **Aura Ledezma-Espinoza:** Software, Investigation, Data curation, Formal analysis, Validation, Resources, Visualization, Funding acquisition, Project administration, Supervision, Writing – original draft, Writing – review & editing. **Esteban D. Avendaño-Soto:** Methodology, Software, Investigation, Formal analysis, Validation, Resources, Writing – review & editing. **Ricardo Starbird-Perez:** Conceptualization, Methodology, Investigation, Formal analysis, Resources, Visualization, Funding acquisition, Project administration, Supervision, Writing – original draft, Writing – review & editing.

## Declaration of Competing Interest

The authors declare that they have no known competing financial interests or personal relationships that could have appeared to influence the work reported in this paper.

## Data Availability

I-CEll raw data (Original data) (Mendeley Data).
